# *Lithocarpus polystachyus* Rehd. ameliorates cerebral ischemia/reperfusion injury through inhibiting PI3K/AKT/NF-κB pathway and regulating NLRP3-mediated pyroptosis

**DOI:** 10.3389/fphar.2024.1365642

**Published:** 2024-09-24

**Authors:** Daifang Liu, Wendan Wu, Tingting Wang, Guiyu Zhan, Yuandong Zhang, Jianmei Gao, Qihai Gong

**Affiliations:** ^1^ Key Laboratory of Basic Pharmacology, Ministry of Education and Joint International Research Laboratory of Ethnomedicine, Zunyi Medical University, Zunyi, China; ^2^ Key Laboratory of Basic Pharmacology of Guizhou Province, Department of Pharmacology, School of Pharmacy, Zunyi Medical University, Zunyi, China; ^3^ Department of Neurology, Affiliated Hospital of Zunyi Medical University, Zunyi, China

**Keywords:** ischemic stroke, *Lithocarpus polystachyus* Rehd., pyroptosis, PI3K/Akt/NF-κB, oxidative stress, inflammation

## Abstract

**Introduction:**

Ischemic stroke (IS) is a serious threat to human life and health, and cerebral ischemia/reperfusion injury (CIRI) exacerbates IS by enhancing neuroinflammation and oxidative stress. Sweet tea (ST) comprises several bioactive components, such as phlorizin, trilobatin, and phloretin, with diverse pharmacological activities. However, it remains uncertain whether ST can confer protection against CIRI. In this study, we aimed to investigate the impact and potential underlying mechanism of ST in the context of CIRI.

**Methods:**

CIRI model were established in male sprague dawley (SD) rats. The neurobehavioral assessment, the volume of cerebral infarction and the morphology of neurons were measured to complete the preliminary pharmacodynamic study. The therapeutic targets and pathways of ST on IS were obtained by protein-protein interaction, molecular docking and Metascape database. The predicted results were further verified *in vivo*.

**Results:**

Our results revealed that ST treatment significantly ameliorated brain damage in rats subjected to CIRI by mitigating mitochondrial oxidative stress and neuroinflammation. Additionally, we identified the PI3K/AKT/NF-κB pathway and the NLRP3-mediated pyroptosis axis as crucial processes, with molecular docking suggested direct interactions between the main compounds of ST and NLRP3.

**Conclusion:**

ST safeguards against CIRI-induced neuronal loss, neuroinflammation and oxidative stress through the inhibition of the PI3K/AKT/NF-κB pathway and the regulation of NLRP3-mediated pyroptosis.

## 1 Introduction

Ischemic stroke refers to a central nervous system disorder caused by impaired blood supply, leading to ischemic and hypoxic necrosis of brain tissue (Baillieul et al., 2022). It is characterized by a high occurrence rate of incidence, mortality and disability, imposing a dreadful incubus on patients and society (Khanna et al., 2021). Up to now, recombinant tissue-type plasminogen activator is the only drug approved by FDA to overcome ischemic stroke (Goncalves et al., 2022). However, its limitations, including a narrow treatment time window, contraindications, and the risk of hemorrhagic transformation, have restricted its benefits for patients (Yuan et al., 2021). Furthermore, restoring blood flow to ischemic brain tissue can exacerbate neuronal death and neurological dysfunction, a condition known as cerebral ischemia-reperfusion injury (CIRI) (Zeng et al., 2022).

CIRI is a critical link in ischemic stroke, involving highly complex pathological processes, amobg which oxidative stress and neuroinflammation playing pivotal roles (Liao et al., 2020; Tuo et al., 2022). Following ischemia and hypoxia, endogenous antioxidant substances in brain tissue were depleted, leading to an accumulation of excessive reactive oxygen species (ROS) (Sies and Jones, 2020; Li et al., 2021). This accumulation induces toll-like receptor 4 (TLR4) activation, which subsequently phosphorylates nuclear factor-κB (NF-κB) (Gao et al., 2020). NF-κB then translocates from the cytoplasm to the nucleus, initiating pro-inflammatory cytokines release involving tumor necrosis factor-alpha (TNF-α), interleukin-1 beta (IL-1β), and interleukin-6 (IL-6) (Yu et al., 2020; Capece et al., 2022). The vicious cycle between oxidative stress and inflammatory responses can trigger the NOD-like receptor pyrin domain containing 3 (NLRP3) inflammasome activation (Han X. et al., 2021; Ma, 2023). The activated NLRP3 inflammasome, in turn, activates cysteinyl aspartate specific proteinase-1 (Caspase-1), which, on one hand, cleaves and activates IL-1β and interleukin-18 (IL-18), thereby mediating and amplifying the inflammatory response (Ising et al., 2019; Ma, 2023). On the other hand, Caspase-1 cleaves Gasdermin D (GSDMD), causing cell membrane perforation and initiating cell pyroptosis, exacerbating CIRI (Frank et al., 2020; Evavold et al., 2021). Therefore, targeting oxidative stress and neuroinflammation regulated by cell pyroptosis holds promise as a therapeutic approach for CIRI.

Sweet Tea (ST), also known as *Lithocarpus Polystachyus* Rehd., is an ethnobotanical medicine primarily found in Guizhou Province, China, and various provinces south of the Yangtze River (Goncalves et al., 2022). It possesses several medicinal properties, including blood sugar reduction, lipid-lowering, anti-inflammatory, antioxidant, and anti-tumor effects (Liao et al., 2020). The main active constituents of ST are phlorizin, trilobatin, and phloretin, and previous literature has suggested that ST can exert neuroprotective effects by inhibiting oxidative stress and inflammatory responses (Shang et al., 2022; Gao et al., 2020). However, there have been no reports on the effects of ST on ischemic stroke. Network pharmacology, a new interdisciplinary field that combines systems biology and computer technology, which analyzes the molecular associations between drugs and diseases from a systems-level perspective (Qin et al., 2022). Meanwhile, natural plants typically have complex active ingredients, multi-target effects, and multiple pathways (Sies and Jones, 2020). Therefore, this study combines network pharmacology, molecular docking technology, and experimental validation to systematically explore and validate the potential mechanisms of ST in preventing ischemic stroke.

## 2 Materials and methods

### 2.1 Materials

The following reagents and antibodies were used in this study: Enzyme-linked immunosorbent assay (ELISA) Kits from Shanghai Renjie Biotechnology Co., Ltd.: IL-6 (RJ15478), IL-18 (RJ15463), IL-1β (RJ15465), TNF-α (RJ16622), Interferon-γ (IFN-γ, RJ15676), Lactate dehydrogenase (LDH, RJ16172), ROS (RJ15780), Superoxide dismutase (SOD, RJ16691), Malondialdehyde (MDA, RJ15503), Glutathione (GSH, RJ29397). Antibodies from Abcam: NLRP3 (ab263899), Apoptosis associated speck-like protein (ASC, ab175449), GSDMD (ab209845), IL-18 (ab191860), IL-1β (ab254360), Caspase-1 (ab286125), Phosphatidylinositol 3-kinase (PI3K, ab1916061), Phosphorylation PI3K (p-PI3K, ab182651), Protein kinase B (AKT, ab8805), Phosphorylation AKT (p-AKT, ab38449), NF-κB (ab16502), Phosphorylation NF-κB (p-NF-κB, Catalog No: ab76302), NF-κB inhibitory protein α (IκB-α, ab32518), Glial fibrillary acidic protein (GFAP, ab7260), Ionized calcium-binding adapter molecule 1 (IBA1, ab178847), Antibodies from Wuhan Sanying Biotechnology Co., Ltd.: Cleaved-Caspase-1 (AF4022), β-actin internal reference (20536-1-AP), GAPDH internal reference (60004-1-lg). Reagents and Standards from Other Sources: TTC staining solution (T8170) from Beijing Solaibao Technology Co., Ltd. Rutin standard (SP8240) from Beijing Solaibao Technology Co., Ltd. Quercetin standard (161009) from Nanjing Zelang Biotech Co., Ltd.

### 2.2 Preparation and analysis of ST

The fresh leaves of *Lithocarpus polystachyus* Rehd. were collected from Guizhou Province in summer, which were deposited in the herbarium and identified by Jianyong Zhang of Zunyi Medical University. The dried ST leaves were ground in a mortar and then transferred to a round-bottom flask. Purified water was added in a 1:20 ratio (plant material to water). After heating and refluxing for 30 min, the mixture was filtered through sterile gauze. This filtration process was repeated twice, and the filtrates were combined. The filtrate was rotary evaporated to obtain an extract, which was then dried in a vacuum dryer. The collected product is the dried powder, which was stored in a −80°C freezer for future use (Gao et al., 2018).

For the analysis of the main representative compounds, trilobatin and phlorizin, in ST based on previous studies, HPLC (High-Performance Liquid Chromatography) was employed. The chromatographic conditions were as follows: Column: Exten-C18 (5 μm, 4.6 × 150 mm), Mobile phase: 1% acetic acid aqueous solution (A)-Methanol (B), Gradient elution (trilobatin: 0–40 min, 70%A-30%B; phlorizin: 0 min, 95%A-5%B; 40 min, 40%A-60%B; 50 min, 0%A-100%B), Maximum wavelength: 286 nm, Injection volume: 10 μL, Column temperature: 30°C, Flow rate: 1 mL/min.

### 2.3 Animal and drug treatment

Male Sprague Dawley (SD) rats weighing 260–280 g from a specific pathogen-free (SPF) environment were obtained from Hunan SJA Laboratory Animal Co., Ltd. (Certificate No: SCXK (Xiang) 2019–0004, Changsha, China). The rats were housed in separate cages (5-6 rats per cage) within an SPF-grade animal facility. During their one-week acclimation period, the animals were kept in rooms with good ventilation, maintained at a constant temperature of 23°C ± 1°C, and a constant humidity of 55% ± 5%. Throughout the acclimation period, the SD rats had free access to food and water. The animal experiments strictly adhered to the ethical guidelines and regulations of the Ethics Committee for Animal Experiments at Zunyi Medical University.

The rats were randomly divided into seven groups using a simple randomization method: the Sham group, middle cerebral artery occlusion (MCAO) group, MCAO + ST 200 mg/kg group, MCAO + ST 400 mg/kg group, and MCAO + ST 600 mg/kg group. ST was administered through intragastric gavage for 7 consecutive days, with one dose per day. Rats in the sham group and the model group were administered intragastrically volume-matched vehiclean equivalent volume of distilled water by intragastric gavage. Twenty-four hours after the last administration, the MCAO model was induced to simulate CIRI.

### 2.4 Animal model

In brief, rats were anesthetized with a 2% solution of pentobarbital sodium (30 mg/kg). Following, the rats were fixed on a board, and the neck area was disinfected with iodine. 2 cm incision was made in the neck area to expose the right common carotid artery, internal carotid artery, and external carotid artery. The external carotid artery was ligated near the common carotid artery. Then, a “V”-shaped incision was made in the common carotid artery, and a filament was inserted until the marked black point. The timer was started, and the incision was sutured. After 2 h of ischemia, the filament was pulled out until the black point was visible, and the excess filament was cut (Gao et al., 2023). The modeling procedure was completed. Throughout the surgery, a constant-temperature heating blanket was used to maintain the rat’s body temperature at around 37°C.

### 2.5 Laser speckle contrast imaging (LSCI) monitoring

To assess the success of the model, cerebral blood flow (CBF) in the whole brain of rats was monitored using a laser speckle blood flow imaging system before modeling, during modeling, and during reperfusion (Gao et al., 2023). The MCAO model was considered successful when CBF decreased to approximately 20% of the preoperative level during modeling and then recovered to around 80% of the preoperative level during reperfusion.

### 2.6 Neurobehavioral assessment

To assess the recovery of rat neurological function, the Longa 5-point method was employed 24 h after reperfusion (Gao et al., 2020). The scoring criteria were as follows: 0 points: No neurological damage; 1 point: Mild neurological impairment, inability to fully extend the paralyzed forelimb on the affected side, walking with a tilted posture; 2 points: Moderate neurological impairment, circling towards the paralyzed side while walking; 3 points: Severe neurological impairment, falling to the paralyzed side when walking, or nearly rotating around the head as the center; 4 points: Inability to walk spontaneously, with signs of loss of consciousness.

### 2.7 Measurement of cerebral infarct volume

After the completion of the modeling process, rats were euthanized, and their brains were carefully and rapidly removed. Subsquently, the brain tissue was sliced into uniform thickness of 5 sections using a blade. These sections were then incubated at 37 C in a constant temperature oven for 60 min with 2,3,5-triphenyltetrazolium chloride (TTC) dye. Following the TTC reaction, the solution was discarded, and a 4% paraformaldehyde solution was added for fixation at room temperature for 2 days, and then TTC-stained slices was quantified by ImageJ software.

### 2.8 Hematoxylin and eosin staining (HE)

The rats underwent cardiac perfusion with PBS (200 mL), followed by perfusion with 4% paraformaldehyde. Subsequently, the brains were carefully and rapidly removed and fixed in 4% paraformaldehyde for 48 h. After dehydration with ethanol, the brain tissues were embedded in paraffin. The paraffin-embedded brain tissues were then cut into 5 μm-thick sections, and the tissue sections were deparaffinized using xylene, and stained with hematoxylin solution for 5 min followed by soaking in eosin solution for 5 min. Finally, the sections were observed and photographed under an optical microscope (BX 43 Olympus, Tokyo, Japan).

### 2.9 Collection and screening of active chemical composition in ST

ST, as a regional ethnic medicine, is not cataloged in various databases. Therefore, this study employed different keywords, including “sweet tea”, “*Lithocarpus Polystachyus Rehd.*,” and others, to search various databases. From the extensive literature related to ST, information on its active components was gathered. Duplicate and structurally unclear compounds were removed, and the data were compiled. These data were then input into the PubChem database (https://pubchem.ncbi.nlm.nih.gov/) to extract the Canonical SMILES names for each component. Subsequently, the SWISS ADME database (https://www.swissadme.ch/) was used to predict the pharmacokinetic properties and drug-likeness of these compound SMILES names. The criteria for selection included “High” gastrointestinal absorption and positive results for Lipinski, Ghose, Veber, Egan, and Muegge filters, with a requirement of “yes” for each and a score of ≥2.

### 2.10 Prediction of potential drug targets and construction of component-target network

SWISS Target Prediction database (http://swisstargetprediction.ch/SwissTargetPrediction) was used to identify the SMILES names of the selected active compounds of ST. The results, in “tsv” format, were downloaded. The duplicate targets and the genes whose credibility value was 0 were removed. Then Cytoscape 3.7.0 software was applied to construct the component-target network.

### 2.11 Acquisition of intersecting target genes

To discover target genes related to ischemic stroke, searches were conducted in the Disgenet database (http://www.disgenet.org/) and GeneCards database (http://www.disgenet.org/) using “Ischemic stroke” as the keyword. An online analysis tool, Venny 2.1 (http://bioinfogp.cnb.csic.es/tools/venny/), was used to create a Venn diagram to visualize the intersection of active components in ST and ischemic stroke-related target genes.

### 2.12 Protein-protein interaction (PPI) network construction

String database (https://string-db.org/) was utilized with a species constraint of “*Homo sapiens*” and a confidence score threshold set at greater than 0.9. This helped in constructing a protein-protein interaction (PPI) network. Nodes that were not connected in the network were hidden. The results, in “tsv” format, were downloaded and imported into Cytoscape 3.7.0 software. A drug-active component-target network was constructed, and the molecular complex detection algorithm (MCODE) plugin was used to identify highly interconnected clusters with default parameters. This comprehensive methodology allowed for the prediction of potential targets for ST and the construction of a network illustrating the relationships among compounds, target proteins, and their interactions.

### 2.13 GO analysis and KEGG pathway enrichment analysis

We conducted a gene ontology (GO) functional enrichment analysis and Kyoto Encyclopedia of Genes and Genomes (KEGG) pathway enrichment analysis on the overlapping targets using the Metascape database (http://metascape.org/gp/index.html). The analysis results were visualized by creating bar graphs and bubble charts using a bioinformatics platform (http://www.bioinformatics.com.cn/). This process aimed to provide a comprehensive understanding of the enriched functions and pathways associated with the intersection targets.

### 2.14 Construction the network of drug ingredients, main pathways, and targets

The active chemical composition in ST, main signal pathways enriched in KEGG analysis, and the corresponding targets of each pathway were imported into Ctyoscape3.7.0 to draw the drug ingredient-main pathway-target network diagram.

### 2.15 Molecular docking

Based on the drug-component-target network results, molecular docking was performed between the main active components of ST (trilobatin, phlorizin, phloretin) and NLRP3. In summary, the 3D crystal structure of the NLRP3 protein (PDB ID: 6NPY) was obtained from the Protein Data Bank (http://www.rcsb.org). The structures of trifolirhizin, root bark glycosides, and quercetin were obtained from the PubChem database and converted to the mol2 format for storage. The target protein and ligands underwent preprocessing, which included steps such as dehydration, hydrogen addition, and atom type assignment, using PyMOL and AutoDock Tools software. Subsequently, molecular docking were performed using AutoDock 4.2. The results were visualized using PyMOL software to gain insights into the interactions between the ligands and the target protein.

### 2.16 Nissl staining

The paraffin-embedded brain tissue sections underwent standard deparaffinization. They were immersed in xylene (55°C, 30 min), followed by double-distilled water to remove excess staining solution. After ensuring clear Nissl bodies under a microscope, the sections were air-dried in a fume hood. Subsequently, neutral resin containing xylene was used to mount the slides. Neuron quantification was performed using the ImageJ 1.80 software.

### 2.17 Immunofluorescence staining (IF)

Brain tissue paraffin sections were subjected to routine deparaffinization, followed by antigen retrieval with citrate buffer using microwave treatment. Non-specific antigen blocking was carried out using goat serum, and the primary antibodies were incubated overnight under 4°C. Subsequently, the corresponding secondary antibodies were added. DAPI staining was performed at room temperature for 5–7 min. Finally, fluorescence was observed using a fluorescence microscope (BX 53 Olympus, Tokyo, Japan), and fluorescence intensity was analyzed by ImageJ software.

### 2.18 Enzyme-linked immunosorbent assay (ELISA)

Blood was obtained from the abdominal aorta, and the serum was collected after centrifuged (3000 × g, 10 min) at 4°C. ELISA were performed following the instructions provided in the respective assay kits to measure the concentrations of IL-6, IL-18, IL-1β, TNF-α, IFN-γ, LDH, ROS, SOD, MDA, and GSH in the serum. Brifly, the standard group, control group and sample group were divided on the 96-well plate, and the standard product, sample diluent, and serum samples diluted 5 times were added in sequence. Then, enzyme-labeled antibodies were added (except control group) and incubated at 37°C for 1 h. After washing for 5 times, add chromogenic solution A and B respectively and incubate at 37°C for 15 min. Finally, add the termination solution. The absorbance value was detected at a wavelength of 450 nm on microplate reader and the sample concentration was calculated according to the standard curve.

### 2.19 Western blot (WB)

In brief, the ischemic penumbra was isolated, and the tissue was completely homogenized on ice. Protein quantification was performed using a BCA assay kit, and the protein samples were denatured by heating. A 10% sodium dodecyl sulfate-polyacrylamide gel electrophoresis (SDS-PAGE) was prepared to separate the proteins, followed by blocking with 5% skim milk powder for 3 h. Subsequently, primary antibodies were used for overnight incubation under 4°C: NLRP3 (1:1,000), GSDMD (1:1,000), NF-κB p65 (1:1,000), p-NF-κB p65 (1:1,000), IL-1β (1:1,000), IL-18 (1:1,000), ASC (1:1,000), Caspase-1 (1:1,000), Cleaved-Caspase-1 (1:1,000), PI3K (1:1,000), p-PI3K (1:1,000), AKT (1:1,000), p-AKT (1:1,000), IκB-α (1:1,000), β-actin (1:10,000), and GAPDH (1:10,000). The following day, HRP-conjugated secondary antibodies were incubated at room temperature for 40 min (1:5,000). Images were exposed and saved, and the grayscale values were analyzed using ImageJ software.

### 2.20 Statistical analysis

All data from this experiment were analyzed using SPSS 18.0 statistical software and are presented as mean ± standard error of the mean (mean ± SEM). Multiple group comparisons were performed using the one-way ANOVA test. If homogeneity of variances was observed, the LSD test was applied. If variances were not homogeneous, the Dunnett’s T3 test was used. A significance level of *P* < 0.05 was considered statistically significant.

## 3 Results

### 3.1 Preparation and analysis of ST

**FIGURE 1:**
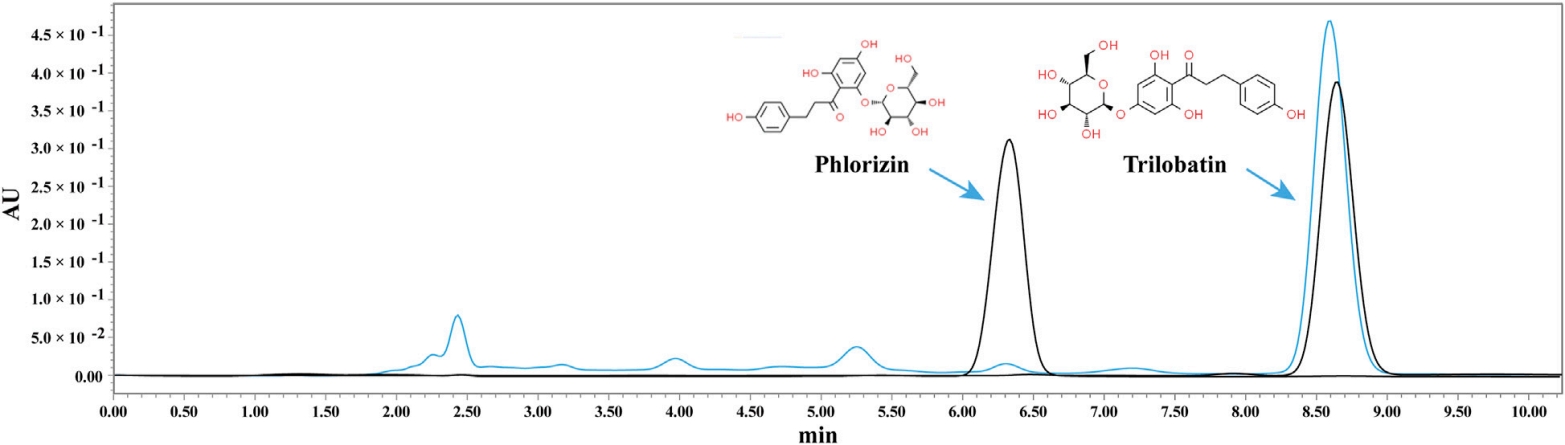
HPLC analysis of ST aqueous extract. The black line represents the standard, and the blue line represents the sample.

ST aqueous extract was prepared from dried ST leaves, and its composition was analyzed using HPLC. Chromatographic results from the HPLC analysis revealed that among the detected peaks, the iconic components of ST, trilobatin and phlorizin, were found (Figure 1).

### 3.2 ST exhibits neuroprotective effect on CIRI

**FIGURE 2:**
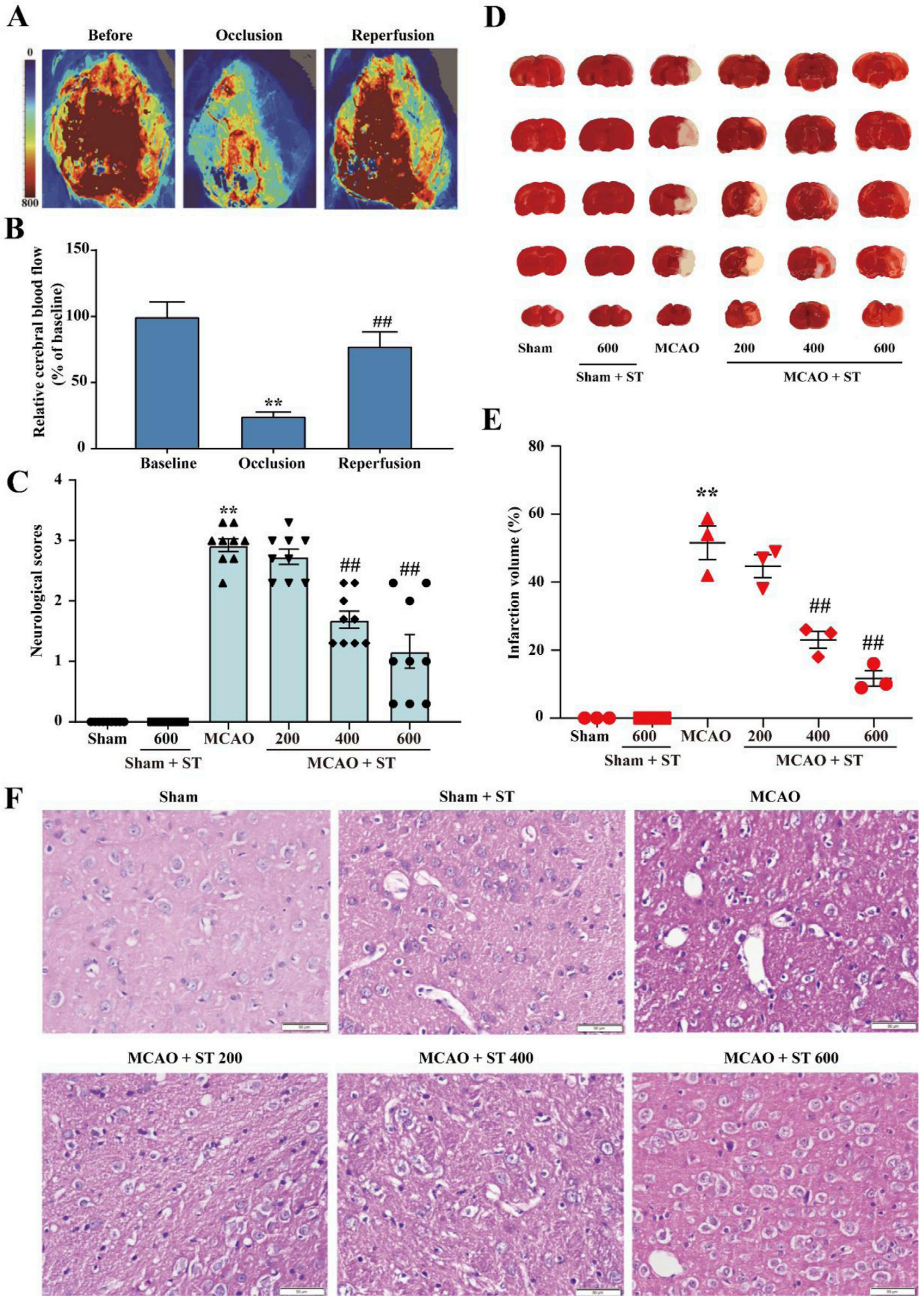
ST significantly improves CIRI in rats. **(A)** Laser speckle imaging representative images. **(B)** Representative images of TTC staining. **(C)** Quantitative analysis of CBF (*n* = 3). **(D)** Results of neurological function scoring (*n* = 9). **(E)** Quantitative analysis of brain infarct volume in each group (*n* = 3). **(F)** HE staining (*n* = 3, Scale bar: 50 µm). Data presented are mean ± SEM. ^*^ *P* < 0.05, ^**^ *P* < 0.01 vs. Sham group; ^#^ *P*< 0.05, ^##^ *P*< 0.01 vs. MCAO group.

To investigate the protective effects of ST on CIRI in rats, neurological function scoring, brain infarct volume measurement, and HE staining were performed at the 24-h time point after successful establishment of the MCAO model. Laser speckle imaging monitoring showed that CBF in rats decreased to approximately 20% of preoperative levels after modeling, and it increased to around 80% of preoperative levels after reperfusion (Figures 2A, B), confirming the successful establishment of the model in this study. Experimental results of neurological function scoring and brain infarct volume measurement demonstrated that the neurological function score and brain infarct volume in the model group were significantly increased compared to the sham surgery group. In comparison to the model group, the administration of ST at 200 mg/kg did not affect the neurological function score and brain infarct volume, whereas the 400 mg/kg and 600 mg/kg groups exhibited significant reductions (Figures 2C–E). Furthermore, HE staining results indicated that after MCAO, there was a significant loss of neurons in the cortical region, or phenomena such as blurred cell boundaries and dark-stained nuclei. However, ST clearly reversed these changes (Figure 2F). These results collectively suggest that ST exhibits neuroprotective effects, significantly attenuating CIRI in rats.

### 3.3 The potential mechanisms of ST in neuroprotection against IS

**FIGURE 3:**
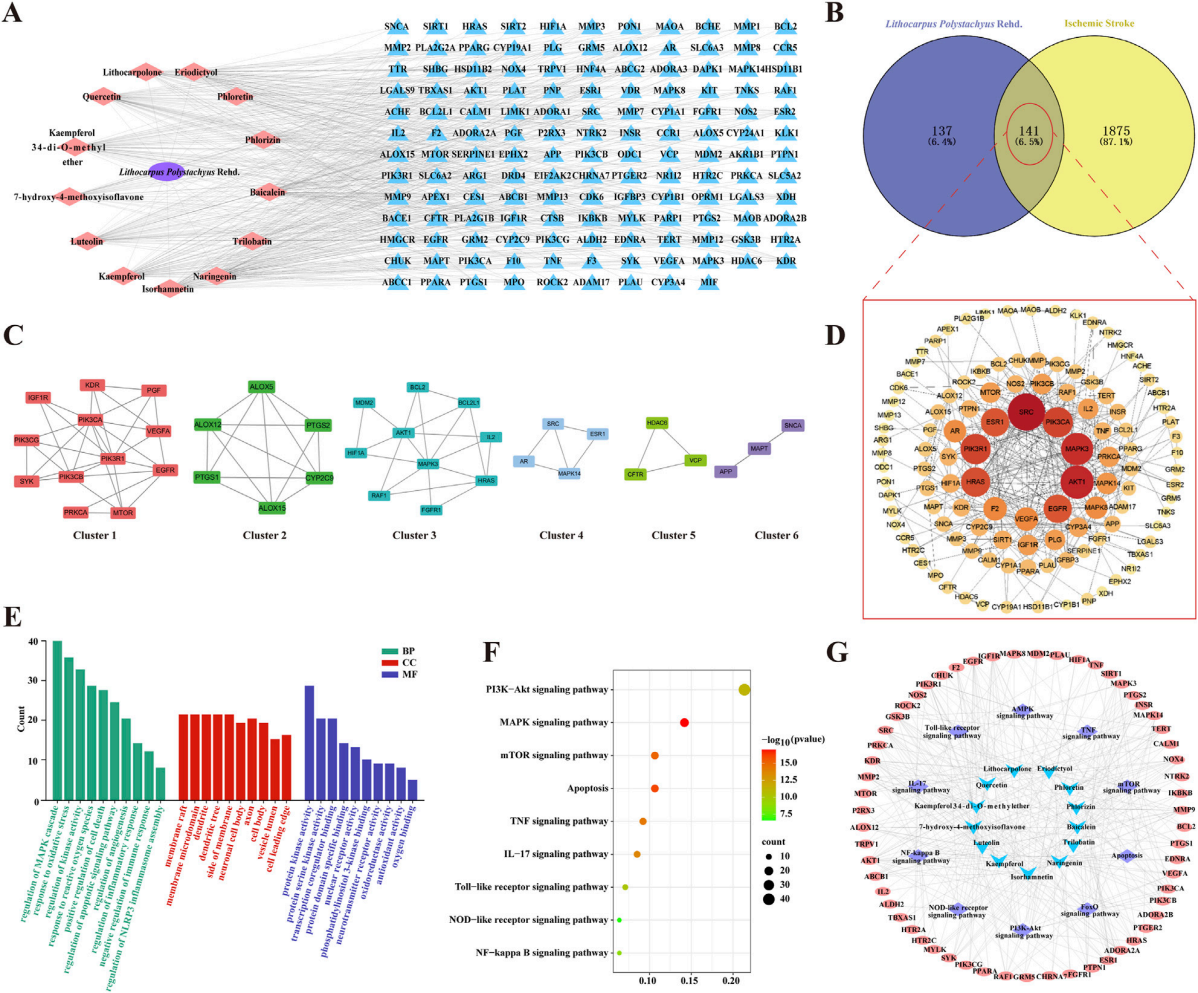
The potential mechanisms of ST in neuroprotection against IS. **(A)** Compound-target network of active ingredients in ST; pink nodes represent active compounds found in ST, and blue nodes represent their corresponding target proteins. **(B)** Venn diagram depicting the overlap between ST targets and IS targets. **(C)** Six clusters obtained from the PPI network. **(D)** PPI network of intersected targets, with the color gradient from yellow to red indicating the significance level of these overlapping targets, with red being the highest and yellow being the lowest. **(E)** Enrichment analysis of GO functions. **(F)** Bubble chart of KEGG pathways. **(G)** Network of drug ingredients, pathways, and targets. Blue nodes represent active compounds found in ST, purple nodes represent signaling pathways these compounds participate in, and pink nodes represent the target proteins of these compounds.

From published literature, a total of 13 potentially active compounds were gathered. These compounds included trilobatin, phlorizin, phloretin, and others (Supplementary Table S1). The SWISS Target Prediction tool was employed to predict target genes for these compounds, resulting in 278 unique target genes after removing duplicates. A compound-target network was constructed and visualized using Cytoscape 3.7.0, comprising 155 nodes and 514 edges (Figure 3A). A total of 2,016 target genes linked to CIRI were obtained from the GeneCards and DisGeNet databases. By comparing disease-related targets with drug-related targets using the Venny 2.1 platform, 141 overlapping targets were identified as core genes for further investigation (Figure 3B). A protein-protein interaction (PPI) network composed of these targets was constructed using the STRING database, resulting in visual graphics and data (Figure 3D). Next, targets in the PPI network was analyzed using MCODE revealed six clusters (Figure 3C; Supplementary Table S2). These results offer new insights into the pharmacological effects of ST to treat CIRI.

Importing core targets into Metascape database for GO analysis: following filtering with a significance level of *P*< 0.05, a total of 216 biological process (BP), 88 cellular component (CC), and 99 molecular function (MF) entries were enriched. In BP analysis, target proteins were primarily involved in processes such as regulation of oxidative stress, inflammatory response, apoptosis, and NLRP3 inflammasome assembly. MF analysis highlighted crucial molecular functions, including protein kinase activity, phosphatidylinositol 3-kinase binding, and antioxidant activity. CC analysis indicated that target proteins were predominantly distributed in membrane rafts, membrane microdomains, dendrites, dendritic spines, membrane periphery, neuronal cell bodies, axons, cell bodies, vesicle lumens, and cell leading edges (Figure 3E). Additionally, a total of 175KEGG signaling pathways were screened out with a significance level of *P*< 0.05. Key pathways such as the NOD-like receptor signaling pathway, PI3K-Akt signaling pathway, TNF signaling pathway, and NF-kappa B signaling pathway were identified as crucial for the anti-IS effects of ST (Figure 3F). Furthermore, our compound-target-pathway network analysis demonstrated that each compound could impact multiple targets and participate in various pathways. Among them, trilobatin, phlorizin and phloretin were identified as key active ingredients in the treatment of IS with ST (Figure 3G).

### 3.4 Molecular docking

**FIGURE 4:**
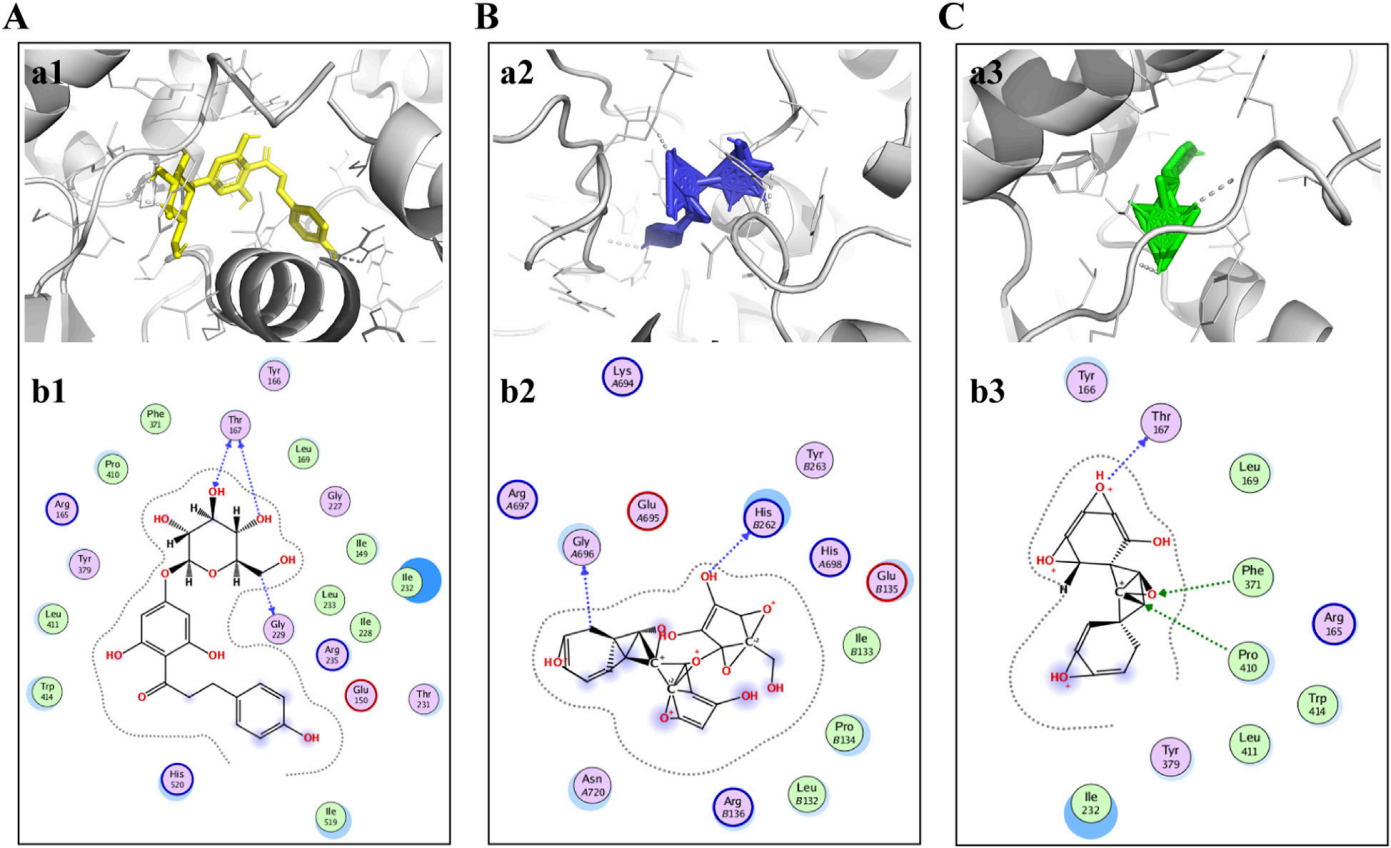
Trilobatin, phlorizin, and phloretin directly bind to NLRP3. **(A)** Trilobatin bound to NLRP3. (a1) Crystal structure of trilobatin (yellow) showing its binding to the docking pocket of NLRP3 (grey). (b1) Amino acid residues involved in the interaction between trilobatin and NLRP3. **(B)** Phlorizin bound to NLRP3. (a2) Crystal structure of phlorizin (blue) demonstrating its binding to the docking pocket of NLRP3 (grey). (b2) Amino acid residues involved in the interaction between phlorizin and NLRP3. **(C)** Phloretin bound to NLRP3. (a3) Crystal structure of phloretin (green) illustrating its binding to the docking pocket of NLRP3 (grey). (b3) Amino acid residues involved in the interaction between phloretin and NLRP3.

As previously mentioned, NLRP3 may be a possible target for ST in the treatment of ischemic stroke. Therefore, molecular coupling method was used to further explore the interaction of key active ingredients trilobatin, phlorizin and phloretin with the target protein NLRP3 (PDBID:6NPY). The results indicated that the binding energies of the three compounds with the target proteins were −5.67 kcal/mol, −5.82 kcal/mol, and −6.28 kcal/mol, respectively. This suggests that the compounds exhibited strong binding affinities with the target proteins. The molecular docking results are detailed below (Figure 4).

### 3.5 ST inhibits NLRP3 activation induced by CIRI and reduces neuronal loss in rats

**FIGURE 5:**
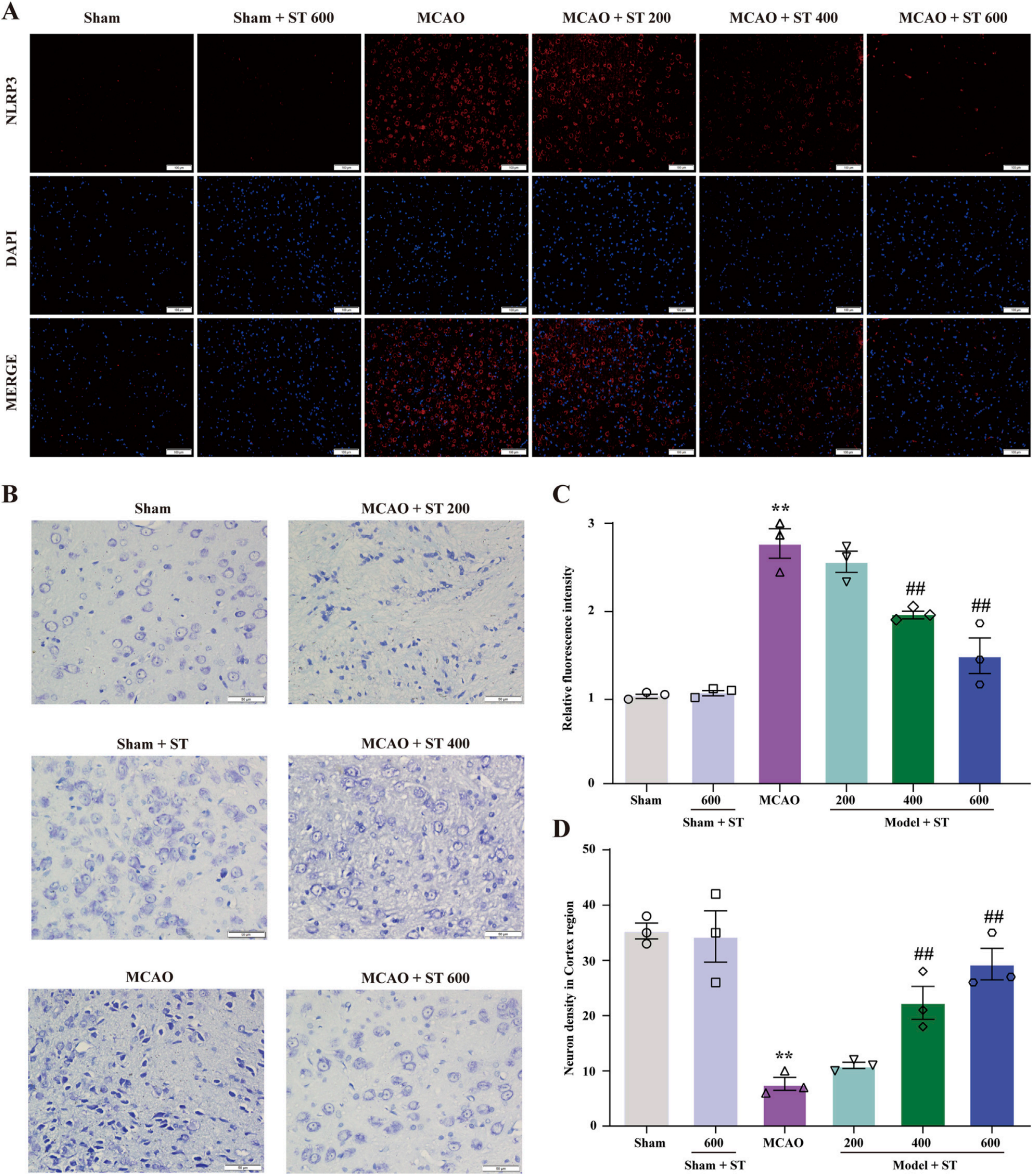
ST reduces CIRI-induced NLRP3 expression and neuronal damage. **(A)** Expression of NLRP3 in the cortical region. **(B)** Nissl staining in the cortical region (scale bar: 50 µm). **(C)** Statistical analysis of relative fluorescence intensity of NLRP3 in the cortical region (*n* = 3). **(D)** Quantification of surviving neurons in the cortical region (*n* = 3). Data presented are mean ± SEM. ^**^ *P* < 0.01 vs. Sham group; ^##^ *P* < 0.01 vs. MCAO group.

To further validate the potential target NLRP3, we conducted immunofluorescence staining (IF). Following CIRI, the NLRP3 fluorescence intensity markedly increased. However, upon treatment with ST, the fluorescence intensity significantly decreased (Figures 5A, C). Furthermore, Nissl staining results showed that in the sham group, neuronal morphology in the cortical region of rats was intact, with full nuclei and clear nucleoli appearing light blue. In contrast, in the model group, Nissl bodies were disorganized, some cell membranes ruptured with vacuoles, nucleoli were deeply stained and shrunken, there was an increase in neuronal loss, and cell degeneration was observed. However, after ST intervention, the 400 and 600 mg/kg dosage groups significantly ameliorated these abnormal changes (Figures 5B, D).

### 3.6 ST dampens pyroptosis induced by CIRI through suppressing the PI3K/AKT/NF-κB pathway

**FIGURE 6:**
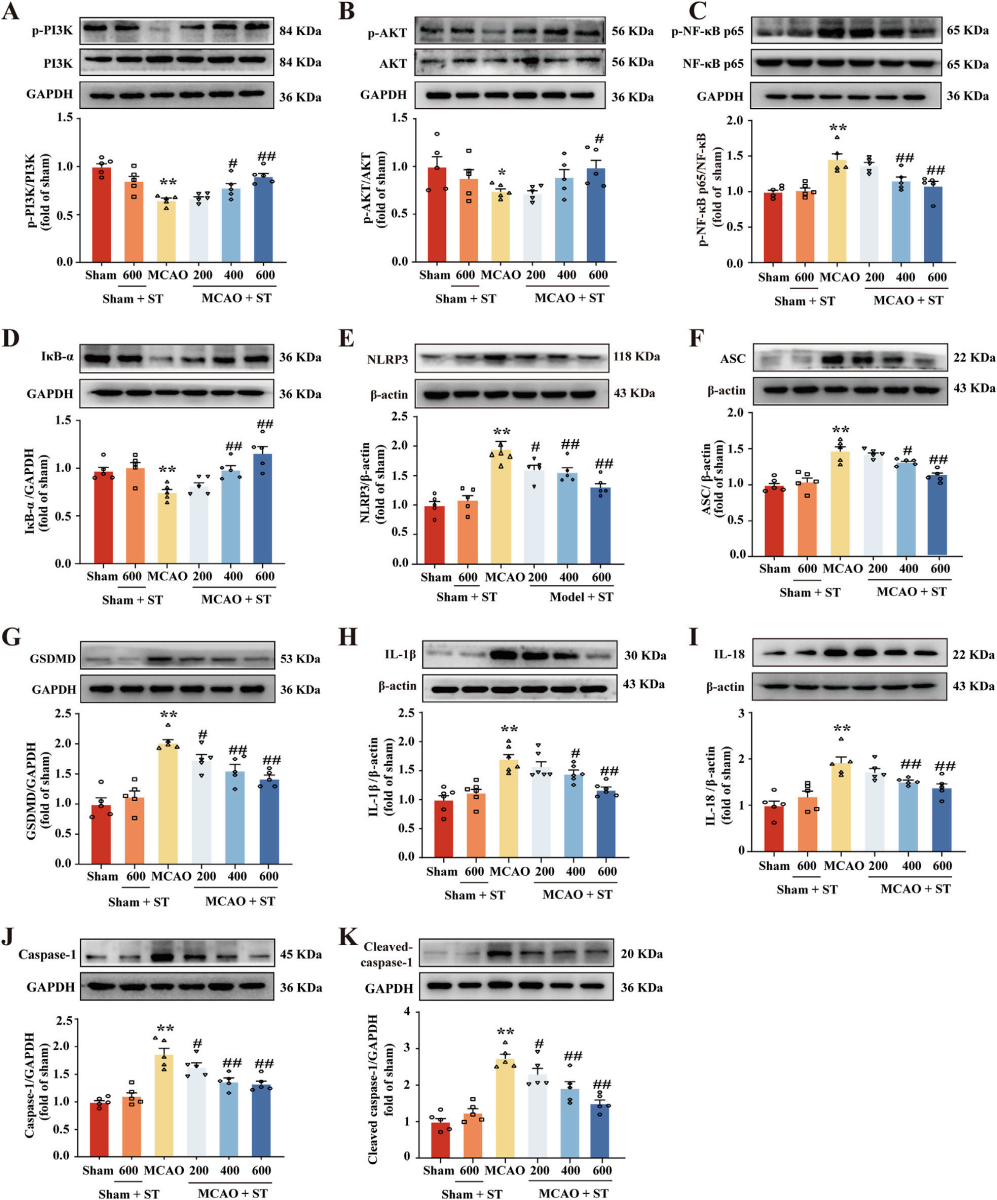
ST dampens pyroptosis induced by CIRI through suppressing the PI3K/AKT/NF-κB pathway. **(A)** Quantitation of p-PI3K (*n* = 5). **(B)** Quantitation of p-AKT (*n* = 5). **(C)** Quantitation of p-NF-κB (*n* = 5). **(D)** Quantitation of IκB-α (*n* = 5). **(E)** Quantitation of NLRP3 (*n* = 5). **(F)** Quantitation of ASC (*n* = 5). **(G)** Quantitation of GSDMD (*n* = 5). **(H)** Quantitation of IL-1β (*n* = 6). **(I)** Quantitation of IL-18 (*n* = 5). **(J)** Quantitation of Caspase-1 (*n* = 5). **(K)** Quantitation of Cleaved-caspase-1 (*n* = 5). ^*^ *P* < 0.05, ^**^ *P* < 0.01 vs. Sham group; ^#^ *P* < 0.05, ^##^ *P*< 0.01 vs. MCAO group.

We utilized WB to validate the findings from the network pharmacology analysis. In rats with CIRI, ASC, GSDMD, IL-18, IL-1β, Caspase-1, Cleaved-Caspase-1, p-NF-κB, and NLRP3 protein expressions were significantly upregulated compared to the sham group. Whereas, after treatment with ST, the expression of these proteins was obviously reduced (Figures 6C, E–K). Additionally, in the model group, p-PI3K, p-Akt, and IκB-α proteins levels were significantly decreased in comparison of the sham group, and this effect was reversed in the ST treatment groups (Figures 6A, B, D).

### 3.7 ST mitigates inflammatory responses and oxidative stress induced by CIRI through inhibiting the activation of gliocyte

**FIGURE 7:**
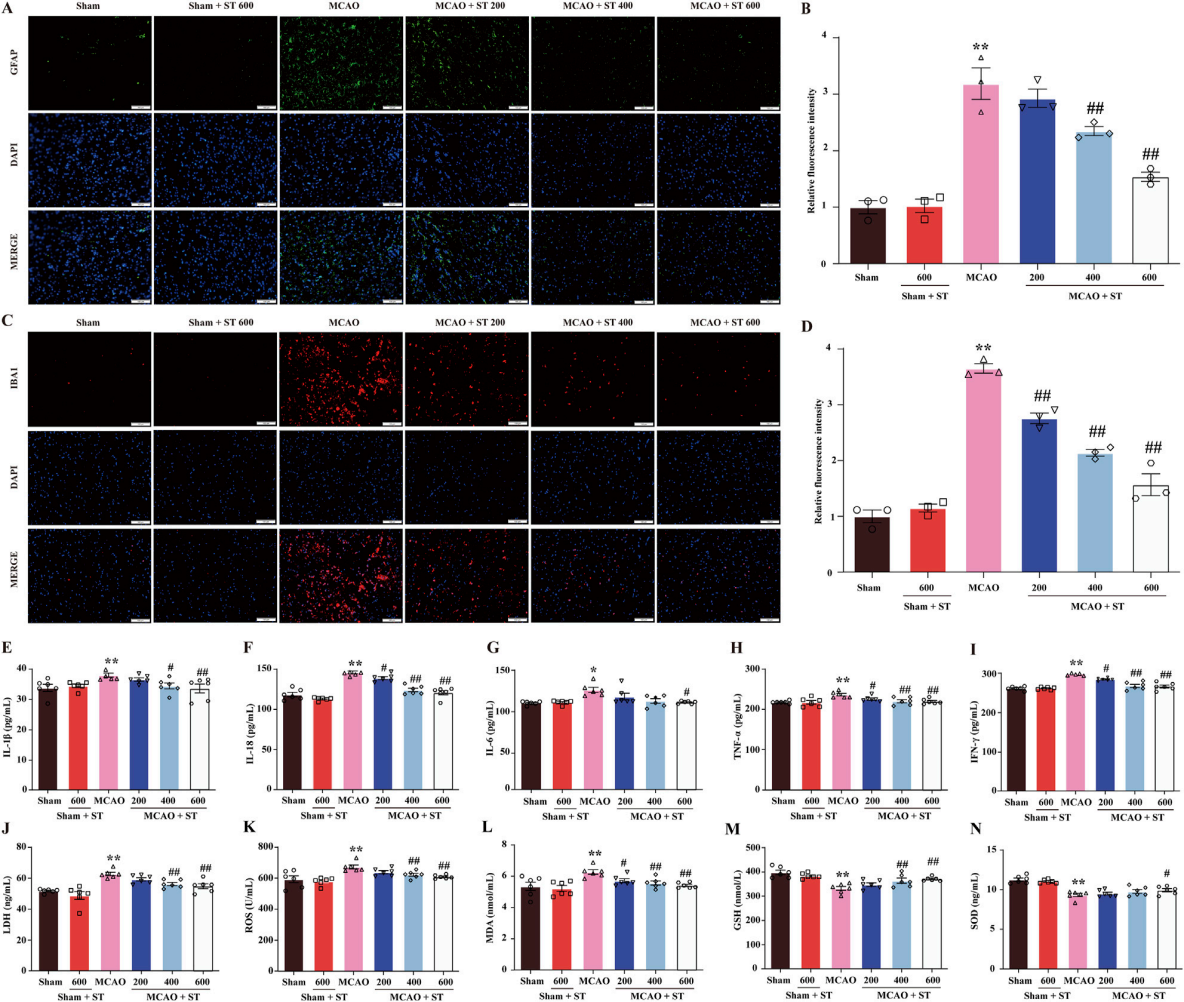
ST mitigates inflammatory responses and oxidative stress induced by CIRI through inhibiting the activation of gliocyte. **(A)** Representative images of GFAP expression (scale bar = 100 µm). **(B)** Representative images of IBA1 expression (scale bar = 100 µm). **(C)** Quantitation of astrocyte fluorescence intensity (n = 3). **(D)** Quantitation of microglia fluorescence intensity (n = 3). **(E)** IL-1β level (*n* = 6). **(F)** IL-18 level (n = 6). **(G)** IL-6 level (n = 6). **(H)** TNF-α level (n = 6). **(I)** IFN-γ level (n = 6). **(J)** LDH level (n = 6). **(K)** ROS (n = 6). **(L)** MDA (n = 6). **(M)** GSH (n = 6). **(N)** SOD (n = 6). Data presented are mean ± SEM. ^**^ *P*< 0.01 vs. Sham group; ^#^ *P*< 0.05, ^##^ *P*< 0.01 vs. MCAO group.

The IF staining was employed to evaluate the fluorescence intensity of GFAP and IBA1 to assess the activation of astrocytes and microglia in the ischemic penumbra of rat brain tissue. The results revealed that in the sham surgery group, both astrocytes and microglia in the ischemic penumbra region were in a resting state. In contrast, in the model group, astrocytes and microglia were significantly activated, exhibiting enlarged cell bodies, increased branching, and a significantly enhanced relative fluorescence intensity. However, intervention with ST reversed these morphological changes (Figures 7A, B) and significantly reduced the relative fluorescence intensity of GFAP and IBA1 (Figures 7C, D). Moreover, the results showed that the levels of IL-6, IL-18, IL-1β, TNF-α, IFN-γ, and LDH in CIRI rats were significantly increased in comparison of sham group. However, following ST intervention, the levels of these pro-inflammatory cytokines were dose-dependently decreased than those of model group (Figures 7E–J). Additionally, it is worth noting that ST significantly improved the levels of oxidative stress-related indicators than those of model group (Figures 7K–N). These findings suggest that ST inhibits the activation of gliocyte, thereby reducing neuroinflammation and oxidative stress following CIRI challenge.

## 4 Discussion

**FIGURE 8:**
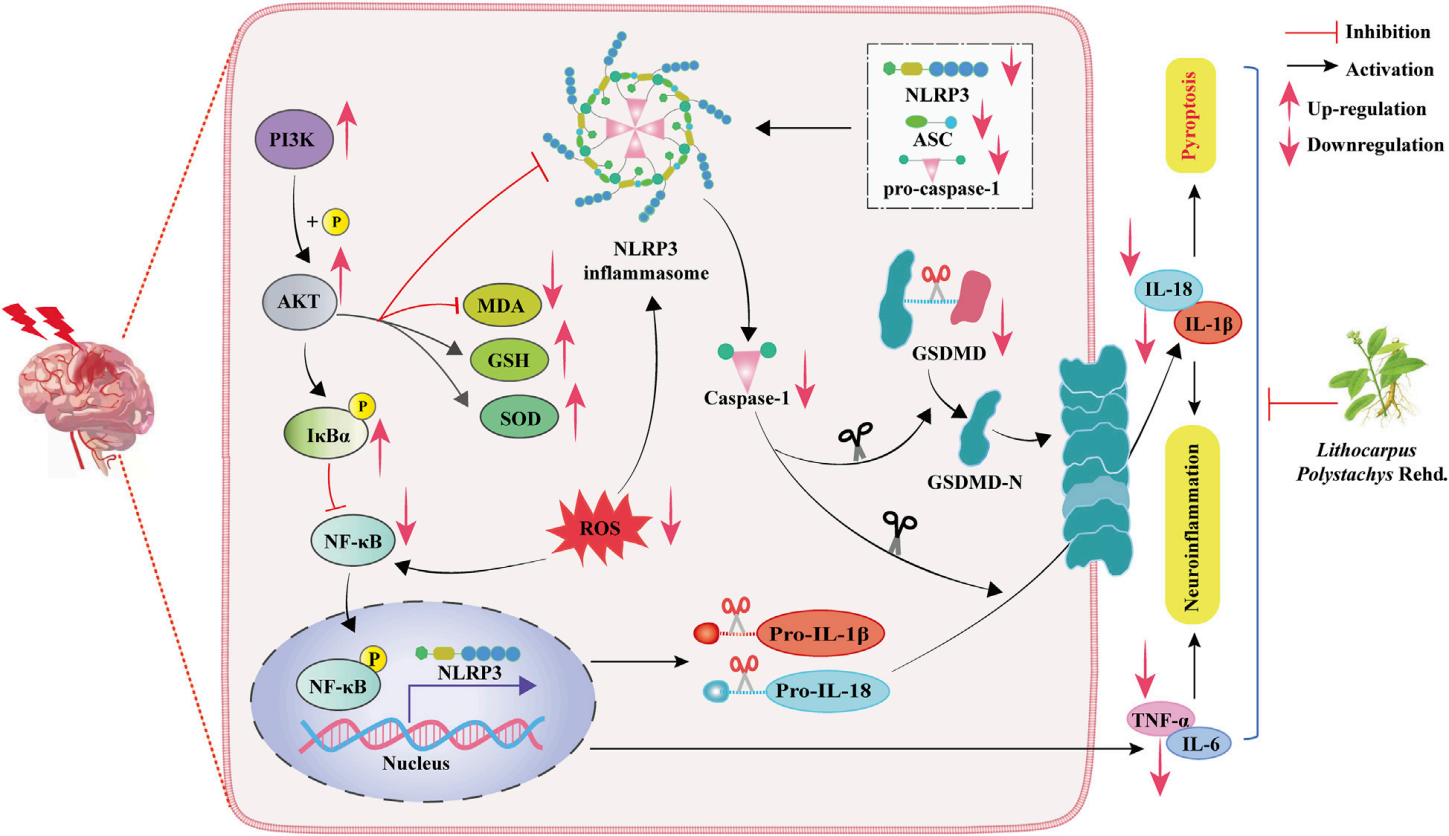
Schematic representation of the molecular mechanisms underlying the protective effects of ST against CIRI.

This study demonstrates that: (1) ST aqueous extract has a significant anti-CIRI effect. (2) ST exerts CIRI protection by modulating the PI3K/AKT/NF-κB and NLRP3-triggered pyroptotic signaling pathways to inhibit oxidative stress and neuroinflammation (Figure 8).

Thus far, due to its complex pathogenesis, there is no ideal treatment for CIRI in clinical practice. Emerging evidence indicates that oxidative stress and neuroinflammation play a pivotal role in worsening neuronal dysfunction and loss following CIRI (Campbell et al., 2019; Qin et al., 2022). Hence, effective drugs for CIRI treatment should exhibit robust anti-neuroinflammatory and antioxidant properties. Prior studies have pinpointed the primary active compounds in ST, including trilobatin, phlorizin, and phloretin, which have demonstrated significant anti-neuroinflammatory and antioxidant effects against various diseases, including IS(Gao et al., 2020; Anan et al., 2022; Li et al., 2023). ST is a medicinal and edible Chinese medicine that integrates the functions of tea, sugar and medicine, and has been approved as a new food raw material with the characteristics of low toxicity and high edible safety (Shang et al., 2022; Liu et al., 2022). So in this study, and considering the pharmacological properties of ST, we embarked on an in-depth exploration of the therapeutic effects of ST on CIRI and its potential targets, with a view to prevent IS through drinking of ST.

Our research results indicate that, through behavioral and neurological assessments, as well as TTC staining, ST can effectively alleviate neurological deficits and cerebral infarction volume, suggesting a protective effect of ST on CIRI. However, the comprehensive and detailed mechanisms of ST are not yet fully understood. Therefore, we employed network pharmacology analysis and molecular docking simulations to predict the potential mechanisms of ST in CIRI. The results suggest that the beneficial effects of ST after CIRI involve the PI3K/AKT/NF-κB pathway and pyroptosis-related pathways, with NLRP3 identified as a key target for ST action. Interestingly, the main active compounds of ST, trilobatin, phlorizin, and phloretin, can directly bind with NLRP3. Earlier study suggests that activation of NLRP3 is elicited within hours of ischemic stroke onset, driving neuroinflammation, ultimately leading to neuronal death (Han B. et al., 2021; Torices et al., 2023). As expected, ST significantly reduced NLRP3 expression and neuronal loss in rats of ischemic penumbra, as confirmed by IF staining and Nissl staining. Moreover, ST reduced the protein expressions associated with the PI3K/AKT/NF-κB pathway and the regulation of pyroptosis following CIRI injury, in alignment with the findings from the network pharmacology analysis. These results indicate that, which indicate that ST may defeat CIRI-induced pyroptosis through modulating the PI3K/AKT/NF-κB pathway. Following brain ischemia, endogenous microglia become activated, secreting inflammation-related cytokines such as IL-1β, IL-18, and TNF-α. Concurrently, activated perivascular astrocytes release numerous vascular permeability factors to exacerbate brain injury (Li et al., 2020; Cramer et al., 2022). In addition, ROS accumulation induced by cerebral ischemia participates in the inflammatory process, resulting in neuronal damage (Wu et al., 2021). Our results show that ST significantly reduced the activation of microglia and astrocytes. Simultaneously, ST significantly lowered serum levels of pro-inflammatory cytokines and oxidative biomarkers, while significantly increased the contents of SOD and GSH in CIRI rats. These findings infer that ST mitigates inflammatory responses and oxidative stress induced by CIRI through inhibiting the activation of gliocyte.

Of note, there are still limitations in current study. Firstly, the precise molecular mechanisms of ST against the acute phase of CIRI, as well as the potential side effects and sustained neuroprotective effects associated with long-term treatment of ST deserve further in-depth exploration. Secondly, more robust data would be needed to be provided to substantiate these findings using a larger sample size, independent replication of results in different settings or with different ischemic models, and data from different layers such as protein, RNA, imaging, etc. Thirdly, this study is limited to an animal model, so human cell-based or 3D models could be further utilized to evaluate the therapeutic potential of ST in humans in the future.

## 5 Conclusion

In summary, the current research results suggest that ST preconditioning evokes robust anti-CIRI effect. ST preconditioning can inhibit NLRP3 inflammasome- mediated pyroptosis by modulating the PI3K/AKT/NF-ΚB signaling pathway, to exert beneficial effect on CIRI-induced neuronal loss, neuroinflammation and oxidative stress.

## Data Availability

The original contributions presented in the study are included in the article/Supplementary Material, further inquiries can be directed to the corresponding author.

## Abbreviations

IS, Ischemic stroke; MCAO, Middle cerebral artery occlusion; CIRI, Cerebral ischemia/reperfusion injury; ST, sweet tea (*Lithocarpus polystachyus* Rehd.); PI3K, phosphatidylinositol-3-kinase; AKT, protein kinase B; NF-κB, nuclear factor-κB; NLRP3, NOD-like receptor family pyrin domain-containing 3; GO, Gene ontology; KEGG, Kyoto Encyclopedia of genes and genomes; PPI, protein-protein interaction.
