# Supplementary material for: Lithocarpus polystachyus Rehd. ameliorates cerebral ischemia/reperfusion injury through inhibiting PI3K/AKT/NF-κB pathway and regulating NLRP3-mediated pyroptosis
Source: Front Pharmacol. 2024 Sep 24;15:1365642. doi: 10.3389/fphar.2024.1365642 (PMC11458458; doi:10.3389/fphar.2024.1365642)
Supplement: Supplementary file 1 [file Table1.docx]

**Supplementary Materials**

**Supplementary Table. S1 Main active ingredients of ST*.***

| Molecule name | PubChem ID | Chemical formula | Relative molecular mass |
| --- | --- | --- | --- |
| Trilobatin | 13188898 | C_21_H_24_O_10_ | 436.409 |
| Phlorizin | 4789 | C_21_H_24_O_10_ | 436.41 |
| Phloretin | 4788 | C_15_H_14_O_5_ | 274.27 |
| Quercetin | [5280343](https://pubchem.ncbi.nlm.nih.gov/compound/5280343) | C_15_H_10_O_7_ | 302.24 |
| Luteolin | [5280445](https://pubchem.ncbi.nlm.nih.gov/compound/5280445) | C_15_H_10_O_6_ | 286.24 |
| Naringenin | 932 | C_15_H_12_O_5_ | 272.25 |
| Eriodictyol | 11095 | C_15_H_12_O_6_ | 288.25 |
| Kaempferol 3，4＇-di-O-methy lether | [5352001](https://pubchem.ncbi.nlm.nih.gov/compound/5352001) | C_17_H_14_O_6_ | 314.29 |
| 7-hydroxy-4＇-methoxyisoflavone | [5280378](https://pubchem.ncbi.nlm.nih.gov/compound/5280378) | C_16_H_12_O_4_ | 268.26 |
| Kaempferol | [5280863](https://pubchem.ncbi.nlm.nih.gov/compound/5280863) | C_15_H_10_O_6_ | 286.24 |
| Isorhamnetin | [5281654](https://pubchem.ncbi.nlm.nih.gov/compound/5281654) | C_16_H_12_O_7_ | 316.26 |
| Baicalein | [5281605](https://pubchem.ncbi.nlm.nih.gov/compound/5281605) | C_15_H_10_O_5_ | 270.24 |
| Lithocarpolone | [78385217](https://pubchem.ncbi.nlm.nih.gov/compound/78385217) | C_31_H_50_O_3_ | 470.73 |

**Supplementary Table. S2 Clusters information of 141 overlapping targets.**

| Cluster | Score | Nodes | Edges | Gene symbol |
| --- | --- | --- | --- | --- |
| 1 | 5.818 | 12 | 64 | PGF, SYK, PIK3CB, MTOR, PRKCA, VEGFA, KDR, PIK3R1, PIK3CG, EGFR, PIK3CA, IGF1R |
| 2 | 5.6 | 6 | 28 | CYP2C9, ALOX5, PTGS1, ALOX15, ALOX12, PTGS2 |
| 3 | 4.444 | 10 | 40 | IL2, AKT1, FGFR1, HIF1A, BCL2L1, MAPK3, HRAS, MDM2, BCL2, RAF1 |
| 4 | 3.333 | 4 | 10 | SRC, ESR1, MAPK14, AR |
| 5 | 3 | 3 | 6 | HDAC6, CFTR, VCP |
| 6 | 3 | 3 | 6 | SNCA, APP, MAPT |
